# CT image segmentation of meat sheep Loin based on deep learning

**DOI:** 10.1371/journal.pone.0293764

**Published:** 2023-11-02

**Authors:** Xiaoyao Cao, Yihang Lu, Luming Yang, Guangjie Zhu, Xinyue Hu, Xiaofang Lu, Jing Yin, Peng Guo, Qingfeng Zhang

**Affiliations:** 1 College of Computer and Information Engineering, Tianjin Agricultural University, Tianjin, China; 2 Tianjin Aoqun Sheep Industry Academy Limited, Tianjin, China; 3 Tianjin Aoqun Animal Husbandry Limited, Tianjin, China; 4 Key Laboratory of Tianjin Meat Sheep Genetics and Breeding Enterprises, Tianjin, China; Shandong Agricultural University, CHINA

## Abstract

There are no clear boundaries between internal tissues in sheep Computerized Tomography images, and it is difficult for traditional methods to meet the requirements of image segmentation in application. Deep learning has shown excellent performance in image analysis. In this context, we investigated the Loin CT image segmentation of sheep based on deep learning models. The Fully Convolutional Neural Network (FCN) and 5 different UNet models were applied in image segmentation on the data set of 1471 CT images including the Loin part from 25 Australian White rams and Dolper rams using the method of 5-fold cross validation. After 10 independent runs, different evaluation metrics were applied to assess the performances of the models. All models showed excellent results in terms evaluation metrics. There were slight differences among the results from the six models, and Attention-UNet outperformed others methods with 0.998±0.009 in accuracy, 4.391±0.338 in AVER_HD, 0.90±0.012 in MIOU and 0.95±0.007 in DICE, respectively, while the optimal value of LOSS was 0.029±0.018 from Channel-UNet, and the running time of ResNet34-UNet is the shortest.

## Introduction

Computed Tomography (CT) is a medical imaging technology that applied X-ray beam to scan an object and converted the received X-ray into the image, which can generate non-overlapping axial section images without incision [1]. As a non-invasive method, CT has been used in animal production and grading [2, 3] over the past years. In CT images, the correlation between animal tissues and X-rays is reflected by Hounsfeld Units (HU) values, and tissues proportions are quantified in different gray levels [4]. Due to the fact that CT imaging technology can classify lesions and other abnormal areas of the scanned object, it is helpful in quickly and accurately diagnosing diseases in medical applications [5].

Image segmentation is an important step in CT image analysis, which has a significant impact on the results of image analysis. The interested areas of image segmentation can be used in automatic disease diagnosis and phenotype measurement. Thus, accuracy of disease diagnosis and phenotype measurement depends on the results of image segmentation. Due to the complexity of internal organs, there are no obvious boundaries between organs and tissues, and it is difficult for traditional methods to correctly segment the internal organs from the background. Deep learning algorithm, which learn to extract important information from the data, has shown excellent performance in image analysis [6], it has been applied in contexts like medical images analysis [7], classification of pepper seed quality [8], detection of tree species [9], animal disease diagnosis [10] and pig carcass analysis [11].

Recent advancements in deep learning have generated breakthroughs in computer vision tasks and showed high performance in image segmentation [12]. Significant progress has been made in the field of human body applications. Xu et al. introduced a semi-supervised segmentation method that utilizes boundary mining models and adversarial learning models for CT images of myocardial infarction [13]. Similarly, Zhang et al. proposed a weighted self-integration framework (SIGN) that incorporates an attention mechanism module to identify lesion areas from a global perspective in fuzzy regions. The method was applied in MRI image segmentation, and achieved the Dice coefficient of 84.14% [14]. Inspired by these studies, we combined deep learning with CT imaging to segment the CT image of sheep. In livestock and poultry breeding, Loin is widely used in breeding research for meat production or meat quality traits [15, 16]. For CT data of meat sheep, Loin segmentation presents an extra challenge due to the absence of obvious boundaries of the region of interest. Deep Learning has proposed the approaches to handle the image segmentation [17].

We selected CT images of meat sheep Loin to explore the technical aspects of deep learning image segmentation, and evaluated the performance of state-of-the-art deep learning modules such as FCN8s, UNet, Attention-UNet, Channel-UNet, ResNet34-UNet, UNet++ in sheep Loin CT image segmentation. These results are of great significance to the living sheep measurement of carcass traits in the future.

## Materials and methods

### Data collection

The dataset came from Tianjin Aoqun Animal Husbandry Co., LTD., the Chinese national core breeding farm for meat sheep, Tianjin, China. The CT Image acquisition were completed in the Production Performance Testing Center of Meat Sheep (China) located in Tianjin Aoqun Animal Husbandry Co., LTD. All animals are treated according to the Experimental Animal Guidelines formulated by the State Council of China.

Animals of Australian White Ram and the Dolper Ram, ranging in age from four months to six months were used in data collection. In September, 2021, a total of 25 healthy ram were scanned, and images of the animals were recorded using scanner system from Neusoft Group Co., LTD(China) and the model of the scanner is NeuViz 16 Classic. The instrument was set at 120 kV/100 mA, with a matrix of 512×512 and an axial thickness of 5.00 mm.

**Fig 1:**
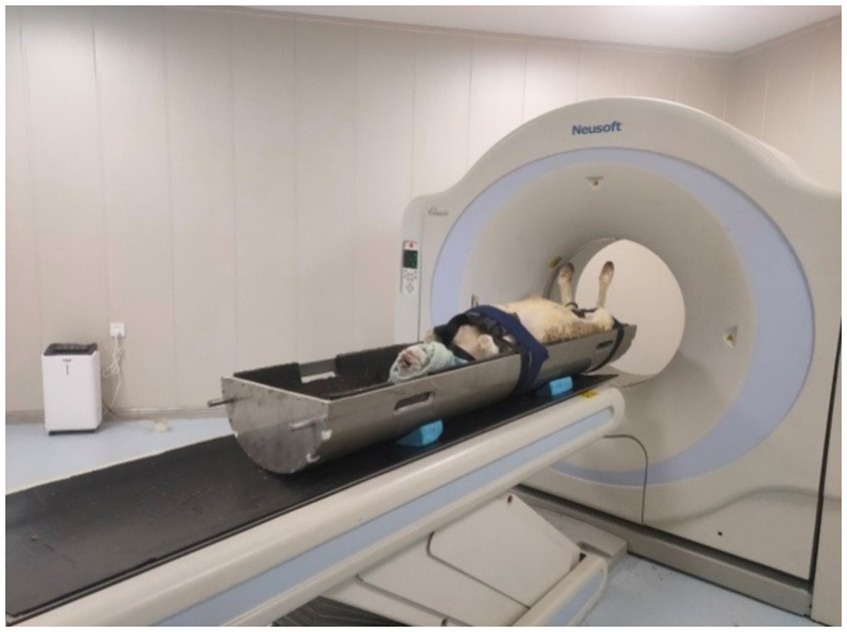
CT scanning of a sheep.

Before scanning, the sheep was given a full-body anesthesia by intravenous injection of 1.0 milliliter of chlorpromazine hydrochloride, and the anesthetized animal was placed in a supine position on the CT scanner platform, bounding in the platform to make sure that the front hooves were curved and the back hooves were naturally extended. The sheep’s head was flipped up and its eyes were covered with eye patch to protect eyes from radiation (Fig 1). When the scanning was completed, the animal was sent to the waiting area to wake up.

**Fig 2:**
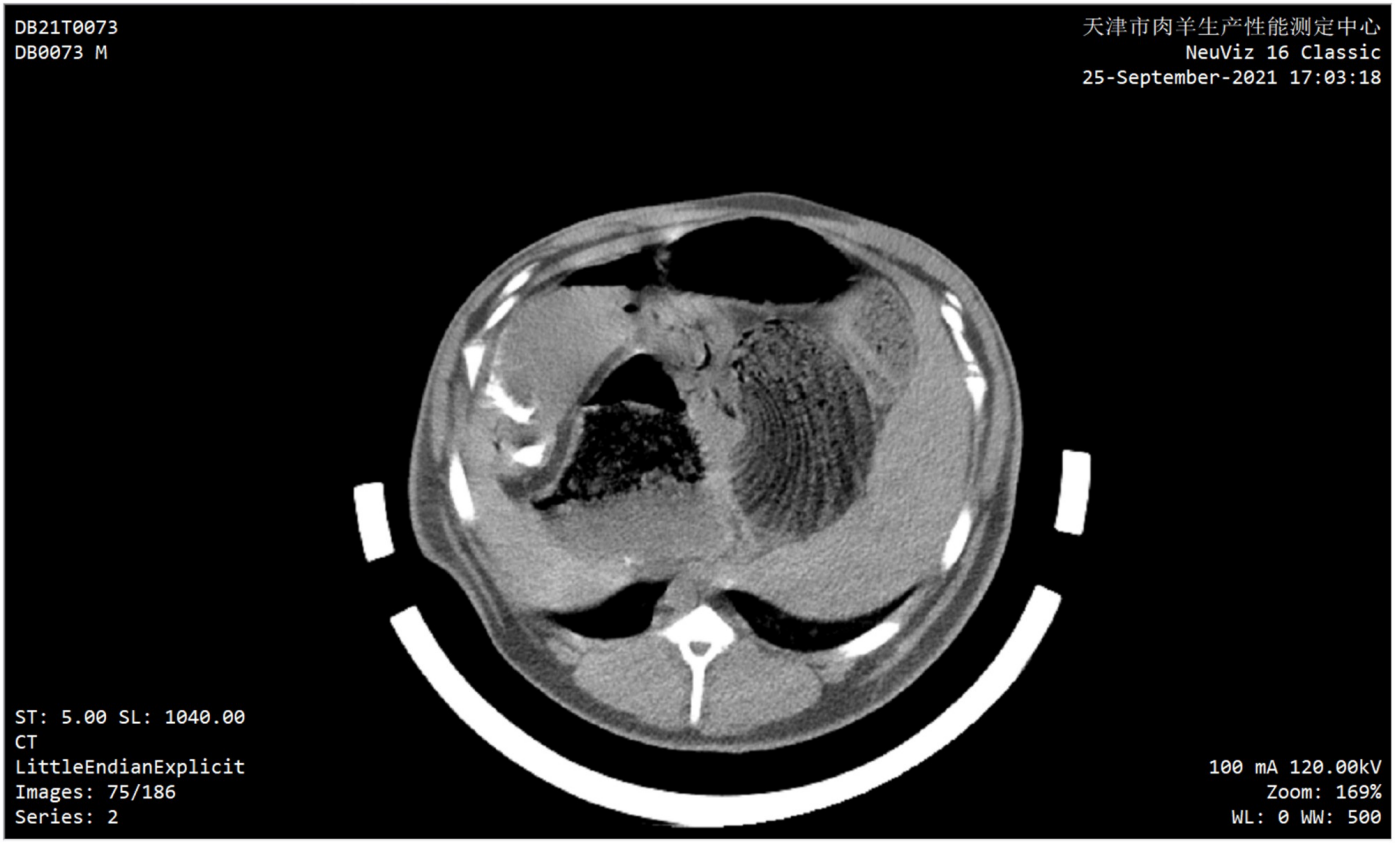
CT image of meat sheep.

When the scanning was completed, all the CT images were downloaded in DICOM format, and the CT intensity was associated with each voxel according to the Hounsfeld scale (Fig 2).

### Data preprocessing

In this study, 25 rams were scanned, and a total of 4508 CT images with the size of 512×512 pixels were obtained. After careful selection, 1471 images including the Loin were picked out to form the experimental dataset.

**Fig 3:**
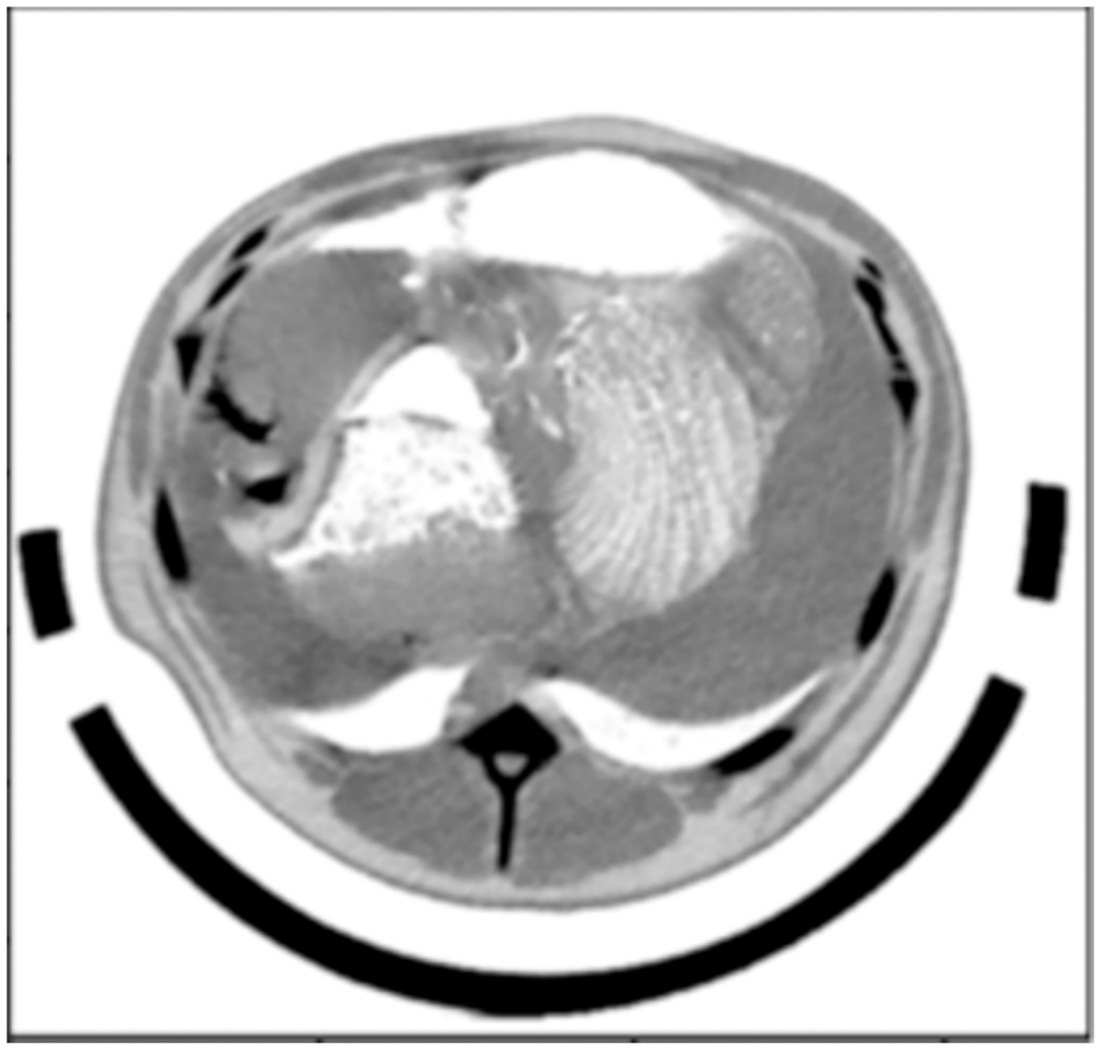
CT image of meat sheep after conversion.

We used MicroDicom open source software to convert DICOM sequence CT image data into JSON file, and finally the JSON file was converted into JPG image file (Fig 3). In order to describe and analyze the feature information of the image, SimpleITK was used to convert the HU value of the images, windowing operations and histogram equalization were also used to improve the contrast between the segmented area and the background in the image [18].

**Fig 4:**
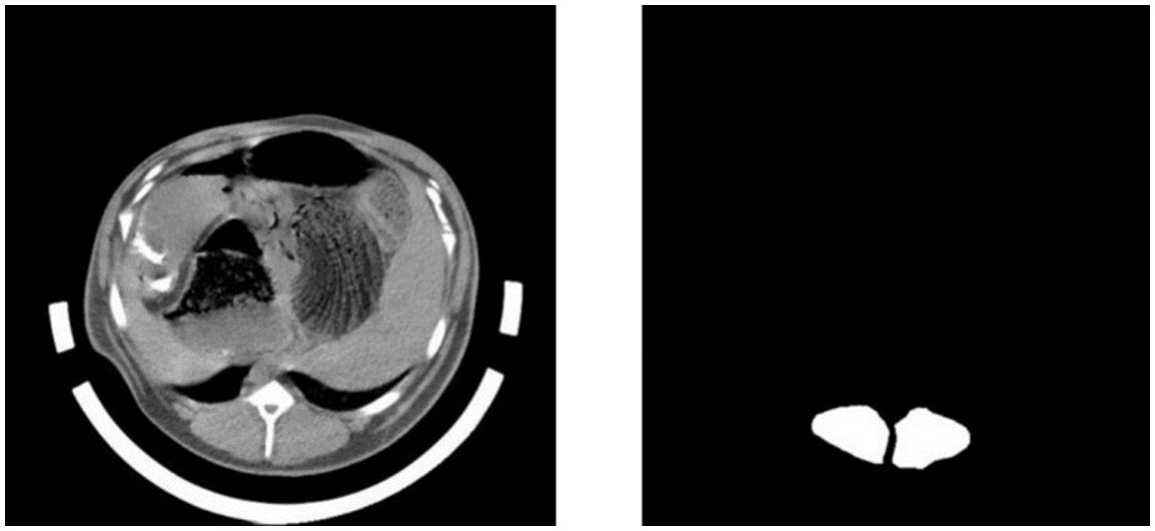
CT image and its annotated image.

The main challenges include the area variation and no visible boundary between the Loin region and the background in CT images. The benchmarks in our study (Ground Truth) were annotated manually by an expert using a semi-automated segmentation tools named LabelMe in Python language open source software library, with binary mask annotations of the CT bed and all internal organs tissues, according to their characteristics and semantic information. The Ground Truth is composed of black pixels for background and white pixels for the Loin (Fig 4).

### Experimental design

To train the deep learning models and evaluate the results, 1471 images from 25 sheep (sheep ID were from 1 to 25) were divided into a training set and validation set, For each time, about one-fifth of 25 sheep were randomly picked out to form the test group according to the method of 5-fold cross validation [19] and the remaining 20 sheep formed the training group. The sheep ID formed test group and training group in a selection were shown (Table 1). CT image of sheep in test group formed test dataset and images of other sheep formed training dataset. The regrouping process of sheep ID and the deep learning images segmentation were repeated 10 times, and the evaluation metrics was calculated for each time. The average and standard deviation of the teach time were calculated to generate the final results.

**Table 1:** A sheep-wise regrouping result in 5-fold cross validation.

Fold	Test group		Training group	
	ID	Num of images	ID	Num of images
1	1, 8, 15, 17, 21	298	2,3,4,5,6,7,9,10,11,12,13,14,15,16,18,19,20,22,23,24,25	1173
2	3, 6, 12, 19, 22	292	1,2,4,5,7,8,9,10,11,13,14,15,16,17,18,20,21,23,24,25	1179
3	5, 7, 14, 18, 23	277	1,2,3,4,6,8,9,10,11,12,13,15,16,17,19,20,21,22,24,25	1194
4	4, 9, 11, 15, 24	301	1,2,3,5,6,7,8,10,12,13,14,16,17,18,19,20,21,22,23,25	1170
5	2, 10, 13, 16, 25	313	1,3,4,5,6,7,8,9,11,12,14,15,17,18,19,20,21,22,23,24	1158

### Deep learning model

UNet have a great power in images analysis and can describe images precisely. Several state-of-the-art deep learning UNet Neural Network models dedicated to image segmentation have been trained in image segmentation. The UNet architectures evaluated were FCN8s, UNet, Attention-UNet, Channel-UNet, ResNet34-UNet, UNet++. A brief review of the main characteristics of each model was described below.

#### Unet

**Fig 5:**
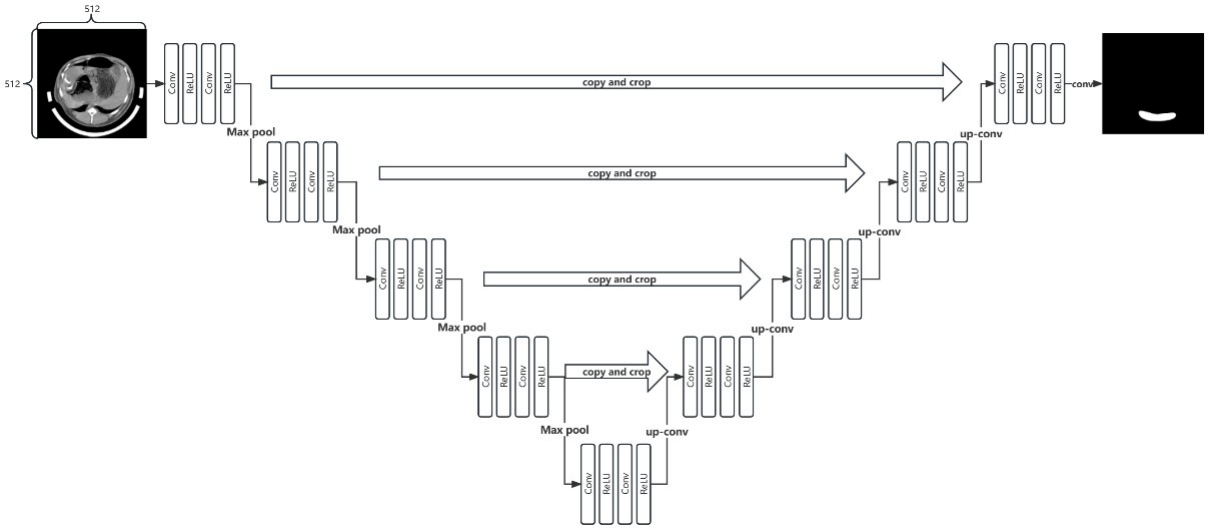
UNet principal architecture.

UNet is a deep learning neural network model for image segmentation. It is a symmetric U-shaped encoder-decoder network, which is combined with a series of convolution, pooling, correction linear units contraction paths and expansion paths [20]. The left half was the encoding path of down-sampling operations, while the right half was the decoding path of up-sampling operations (Fig 5).

The encoding path in UNet extracts the main features from image data and consists of four modules, each of which includes two 3×3 convolution operations to extract local features of the image. After the convolution operation, the image is compress into 2×2 pixels, and then the corrected linear unit filters the relevant features of the image. UNet uses the ReLU activation function in the correction linear unit to calculate the correction value to reduce the disturbance that may occur in the translation, rotation and other operations of the image to prevent over fitting. The decoding path in UNet recovers the image data compressed by the shrink path. When the image is restored to its original size, the image details are rearranged according to the core features, and the interested parts in the image are accurately located. Different from the contraction path, the expansion path replaces the 2×2 max pooling in the down-sampling part with a 2×2 deconvolution, and the number of feature channels is halved by the up-sampling of each layer, while the image size is expanded by 2×2 pixels after the convolution of each layer. In the last step of up-sampling, UNet uses a 1×1 convolution kernel to map the image with 64 feature channels to two feature channels. These two feature channels output the image target part and the image background part respectively.

#### FCN

Based on the classification networks of AlexNet, VGGNet and GoogLeNet, Fully Convolutional Networks (FCN) changes the fully connection layer into fully convolutional layer. In addition, deconvolution is used in the up-sampling process to solve the problem of image size reduction caused by convolution and pooling, and a skip structure is adopted to integrate deep features with shallow features [21]. FCN can accept the input of any size image, increase the image size, and output accurate results.

#### UNet++

UNet++ adds a dense skip connection to UNet deep learning neural network, which can produce a more flexible feature fusion scheme and make the feature learning more efficientive. At the same time, UNet++ reduces training errors caused by deep-level problems of unknown networks by integrating UNet with different depths [22].

#### Attention-Unet

Attention-UNet uses the attention-gate module to highlight important relevant features of images and suppress irrelevant features. In this model, hard-attention is replaced by soft-attention, and the attention module is integrated into the skip connection and up-sampling module of UNet network to implement spatial attention mechanism [23].

#### ResNet34-Unet

ResNet34-UNet uses lightweight network, where ResNet34 replaces the feature extraction layer in the original UNet and introduces a large number of skip connections, which can improve the accuracy of image segmentation while maximizing the combination of image deep semantic information. ResNet34 has the advantages of fast convergence, low training data volume and easy training, effectively reducing the problem of gradient disappearance and non-convergence of LOSS [24].

#### Channel-Unet

Channel-UNet is an end-to-end convolutional neural network, which takes UNet as the main framework and adds spatial Channel convolution operations in the up-sampling and down-sampling modules. It effectively maps the pixel information in the high-level feature map and achieves the fusion of multi-scale feature information [25].

### Pixel accuracy

Pixel accuracy is defined as the probability of pixels correct classification in the category. Generally, the number of pixels correctly predicted is divided by the total number of pixels in the category. Its formula is defined as

$$PA=\frac{TP+TN}{TP+TN+FP+FN}$$ (1)

TP represents the number of samples correctly predicted as positive samples; TN represents the number of samples correctly predicted as negative samples; FP represents the number of samples incorrectly predicted as positive samples; FN represents the number of samples incorrectly predicted as negative samples.

### Evaluation metrics

Many evaluation criteria have been proposed to assess the performance of deep learning image segmentation, in order to have a comprehensive comparison about our experiments, we selected LOSS, AVER_HD, MIOU, DICE to analyze deep learning results.

#### LOSS

LOSS represents the degree of inconsistency between the predicted value and the real value, and the stable LOSS values is preferred [26]. In this paper, binary cross entropy loss is used as a measurement standard, and its formula is

$$L=-\frac{1}{N}\sum_{i=1}^{N}\left[y_{i}\;\text{log}\left(p_{i}\right)+\left(1-y_{i}\right)\text{log}\left(1-p_{i}\right)\right]$$ (2)

Where, N is the total number of samples, *y*_*i*_ is the category of the ith sample, and *p*_*i*_ is the predicted value of the ith sample. For LOSS, smaller value means better result.

#### AVER_HD

AVER_HD is the average Hausdorff distance, which calculates the similarity between the real value and the predicted value, and is more sensitive to the segmented boundary parts. Smaller AVER_HD value means higher similarity between the predicted value and the real value [27]. For sets X = {*x*_1_,*x*_2_,…,*x*_*n*_} and Y = {*y*_1_,*y*_2_,…,*y*_*n*_} in Euclidean space, the bi-directional Hausdorff distance between set X and set Y is:

$$d\_H(X,Y)=max(h\left(X,Y\right),h\left(Y,X\right))$$ (3)

Where h(X, Y) = *max*_*x*∈*X*_ min_*y*∈*Y*_ ||x − y||, h(X, Y) is the unidirectional Hausdorff distance from X to Y and h(Y,X) is the unidirectional Hausdorff distance from Y to X.

#### MIOU

MIOU represents the average segmentation accuracy of prediction, which calculates the IOU value on each category. MIOU is defined as,

$$MIOU=\frac{1}{k+1}\sum_{i=0}^{k}\frac{p_{ii}}{\Sigma_{\dot{J}=0}^{k}p_{ij}+\Sigma_{j=0}^{k}p_{ji}-p_{ii}}$$ (4)

here, *P*_*ij*_ represents the number of predicted *j* whose true value is *i*, and *k*+1 is the number of categories. *P*_*ij*_ and *P*_*ji*_ represent false positive and false negative respectively [28]. Larger value of MIOU means better accuracy.

#### DICE

DICE means the similarity between the predicted value and real value, which is defined as

$$DICE=\frac{2\left|X\cap Y\right|}{\left|X\right|+\left|Y\right|}$$ (5)

Where, |X| is the ground_truth, |Y| is the predict_mask, |*X* ∩ *Y*| is the overlapping area of the real background and the predicted background, and |*X*| + |*Y*| is the sum of all regions containing the overlapping area of the real background and the predicted background [29]. For DICE, larger value means better result.

## Experimental results

### Computer system

The calculations were conducted on a computer equipped with 12th Gen Intel Core i7-12700F CPU, 32GB memory, NVIDIA GeForce RTX 3060 Display card (12GB GPU video memory). The operating system was Microsoft Windows, and the software running environment included PyCharm Community Edition 2020.2.1x64 compiler, Anaconda 4.10.3 Python development package, TensorFlow-GPU 2.4.0 and Keras 2.4.3 deep learning open source libraries. The GPU based software running environment was CUDA 11.0 and CUDNN 8.0.1.

### Model training results and comparison

#### Experimental settings

The number of training iterations was 300 and the learning rate was set to 1×10^−4^, the batch size was set to 4, and GPU training mode was used.

#### Model comparison

**Fig 6:**
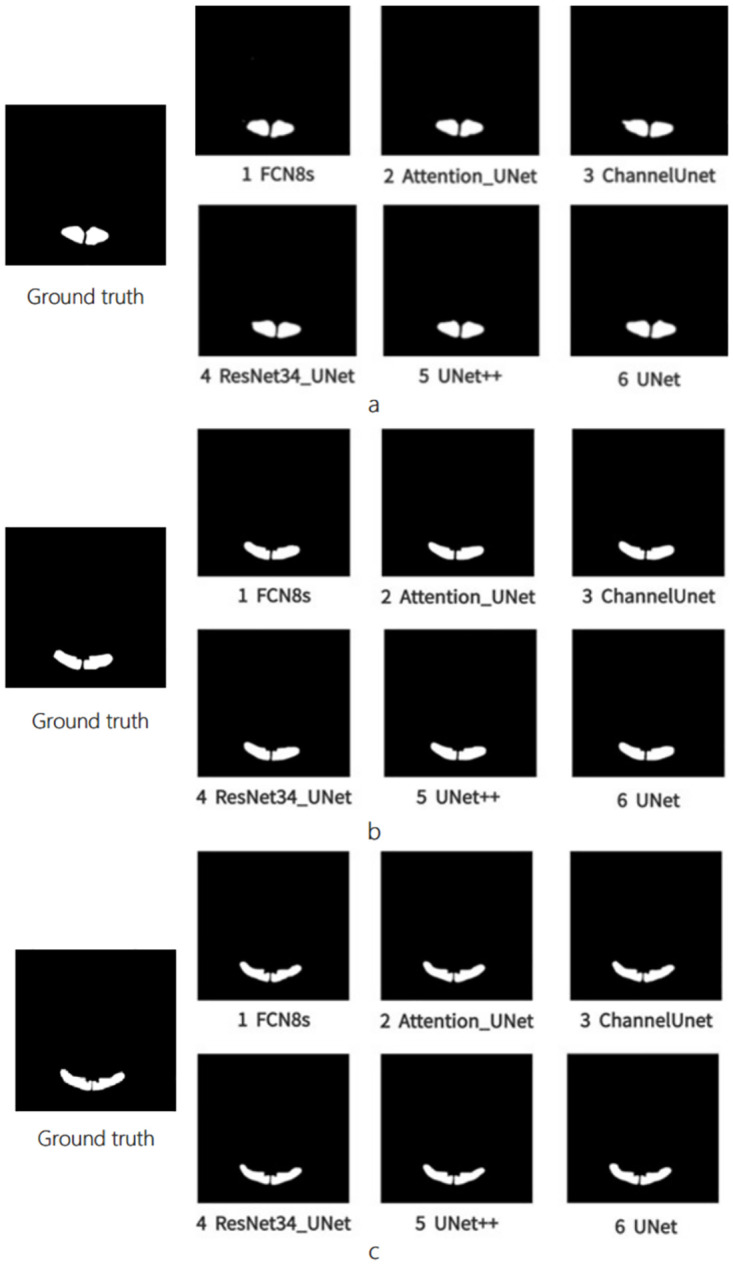
Prediction results from a sheep ’s loin CT images, (a) Results of CT images in the front section, (b) Results of CT images in the middle section, (c) Results of CT images in the rear section.

**Fig 7:**
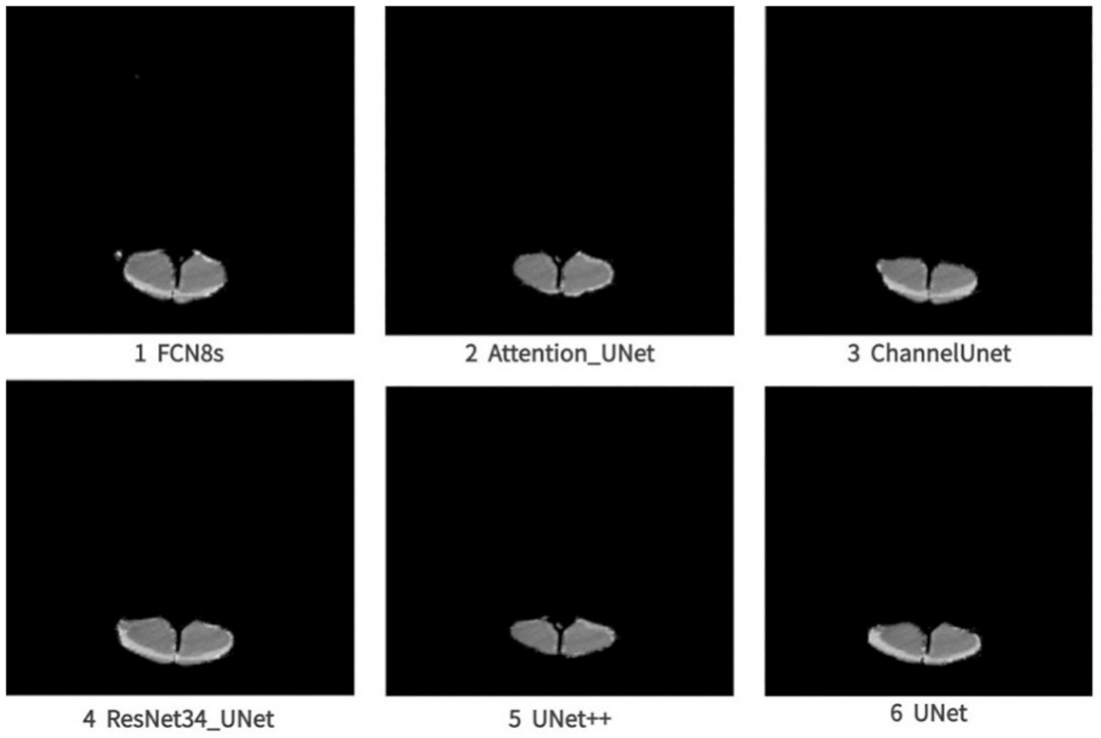
The result graph after segmentation.

In experiments, we ran FCN8s, Attention-UNet, Channel-UNet, ResNet34-UNet, UNet++ and UNet to process sheep CT image segmentation (Fig 6). In the figures, areas marked with white color were the predicted areas of the Loin in CT images. From the figures, we can find that the outline, shape and area of the predicted results of the Ground Truth were similar, which indicated that the performances of these models were excellent. With the predictive results of deep learning algorithm, we can obtain the segmentation results of Loin area using bit operation between predictive areas and the original image (Fig 7). From the results, slight differences could be found among the results.

For each model, the training of the five-fold cross validation deep learning image segmentation was repeated 10 times and the average value and standard deviation were calculated as the result. Pixel accuracies of the 6 models were shown (Table 2). From this table, we can find that Attention-UNet outperformed other five methods with the accuracy of 0.999±0.009. The second best result was 0.998±0.015, which was generated by UNet++. As far as running time was concerned, the time of ResNet34-UNet was the shortest, and its values was 22.078±0.368h.

**Table 2:** Accuracies and Running time in CT image segmentation (Mean ± Standard Deviation).

Model	Pixel accuracy	Trainable parameters	inference time of a single CT image	inference time of an entire CT image	Entire Running Time
FCN8s	0.981±0.105	*18643713*	4.489±0.013s	202±0.013s	28.056±0.468h
Attention-UNet	**0.999±0.009**	34878573	**3.000±0.011s**	**135±0.011s**	51.456±0.858h
Channel-UNet	0.997±0.054	49153601	8.756±0.025s	394±0.025s	56.878±0.948h
ResNet34-UNet	0.996±0.076	21656897	4.222±0.015s	190±0.015s	**22.078±0.368h**
UNet++	*0*.*998±0*.*015*	47190724	*4*.*000±0*.*010s*	*180±0*.*010s*	49.056±0.818h
UNet	0.996±0.090	**7765442**	7.467±0.053s	336±0.053s	*26*.*278±0*.*438h*

Results of evaluation metrics like AVER_HD, MIOU, DICE and LOSS of 6 deep learning models were shown (Table 3). These values were also provided in Mean ± Standard Deviation after 5-fold cross-validation evaluation. From Table 3, we could find that Attention-UNet outperformed other five methods on metrics of AVER_HD, MIOU and DICE with values of 4.391±0.338, 0.900±0.012 and 0.947±0.007 respectively. UNet performed second best among these models on AVER_HD (4.597±0.347), MIOU (0.898±0.012) and DICE (0.946±0.006). For LOSS, Channel-UNet performed best with values of 0.029±0.018.

**Table 3:** Evaluation metrics comparisons of different models (Mean ± Standard Deviation).

Model	AVER_HD	MIOU	DICE	LOSS
FCN8s	4.877±0.371	0.885±0.018	0.935±0.001	*0*.*192±0*.*117*
Attention-UNet	**4.391±0.338**	**0.900±0.012**	**0.947±0.007**	0.199±0.115
Channel-UNet	4.651±0.278	0.896±0.011	0.945±0.006	**0.029±0.018**
ResNet34-UNet	4.734±0.426	0.890±0.014	0.942±0.007	0.387±0.093
UNet++	4.717±0.143	0.886±0.022	0.938±0.025	1.998±0.430
UNet	*4*.*597±0*.*347*	*0*.*898±0*.*012*	*0*.*946±0*.*006*	0.246±0.098

Note: for each column, the optimal was marked with bold and the suboptimal was marked with italic.

**Fig 8:**
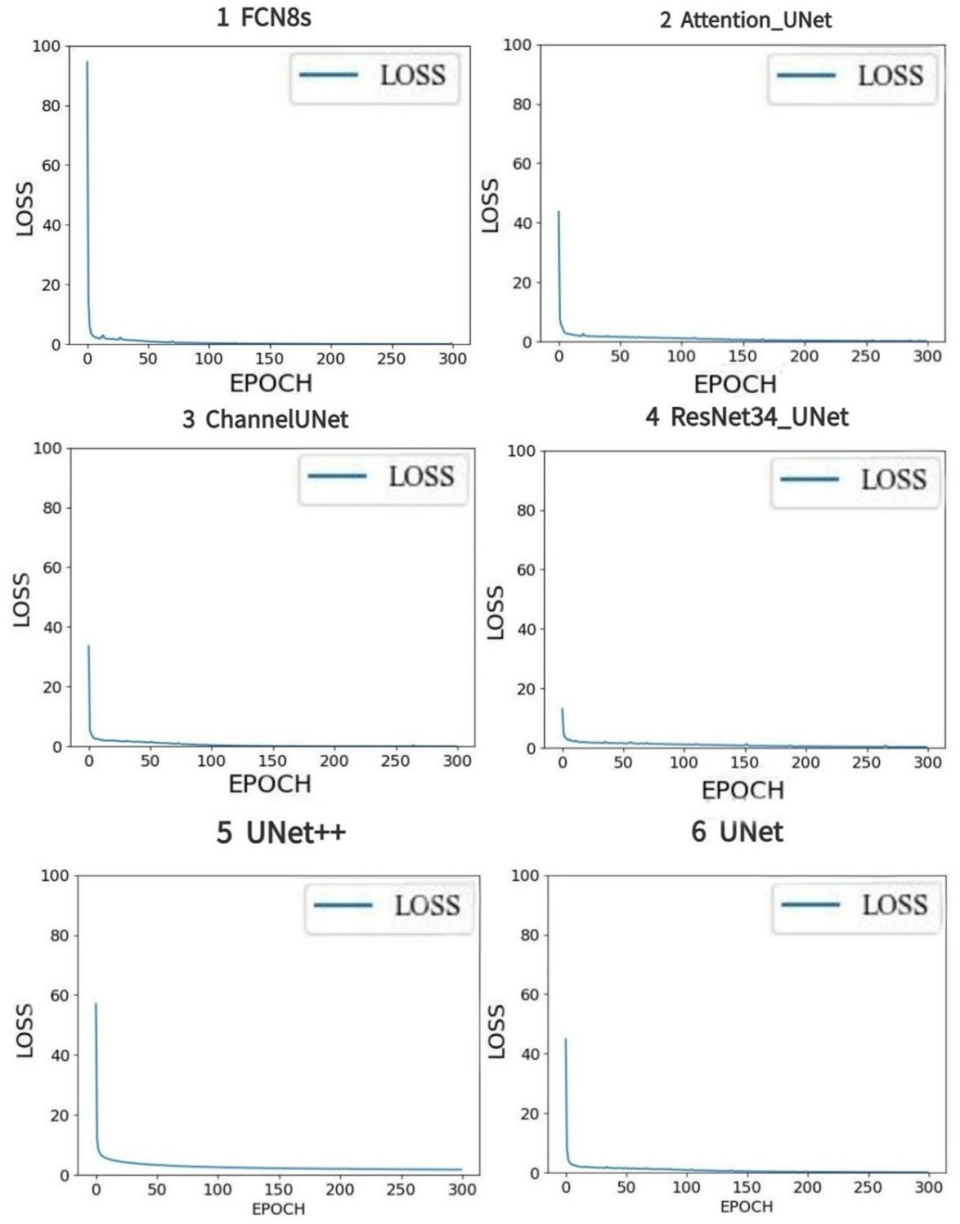
LOSS results of six deep learning models.

The value of LOSS function in the training of 6 deep learning models were shown (Fig 8), from these figures, we could find that the values of LOSS function gradually became stable with the progress of training. However, there were slight differences among the convergence of the LOSS function obtained from different models and the values of LOSS function from FCN8s, Unet, UNet++ converges relatively faster. The largest LOSS value was close to 2, which was generated by UNet++ and the smallest one was generated by Channel-UNet and the value approached 0.03. For LOSS, Channel-UNet outperformed other five models.

**Fig 9:**
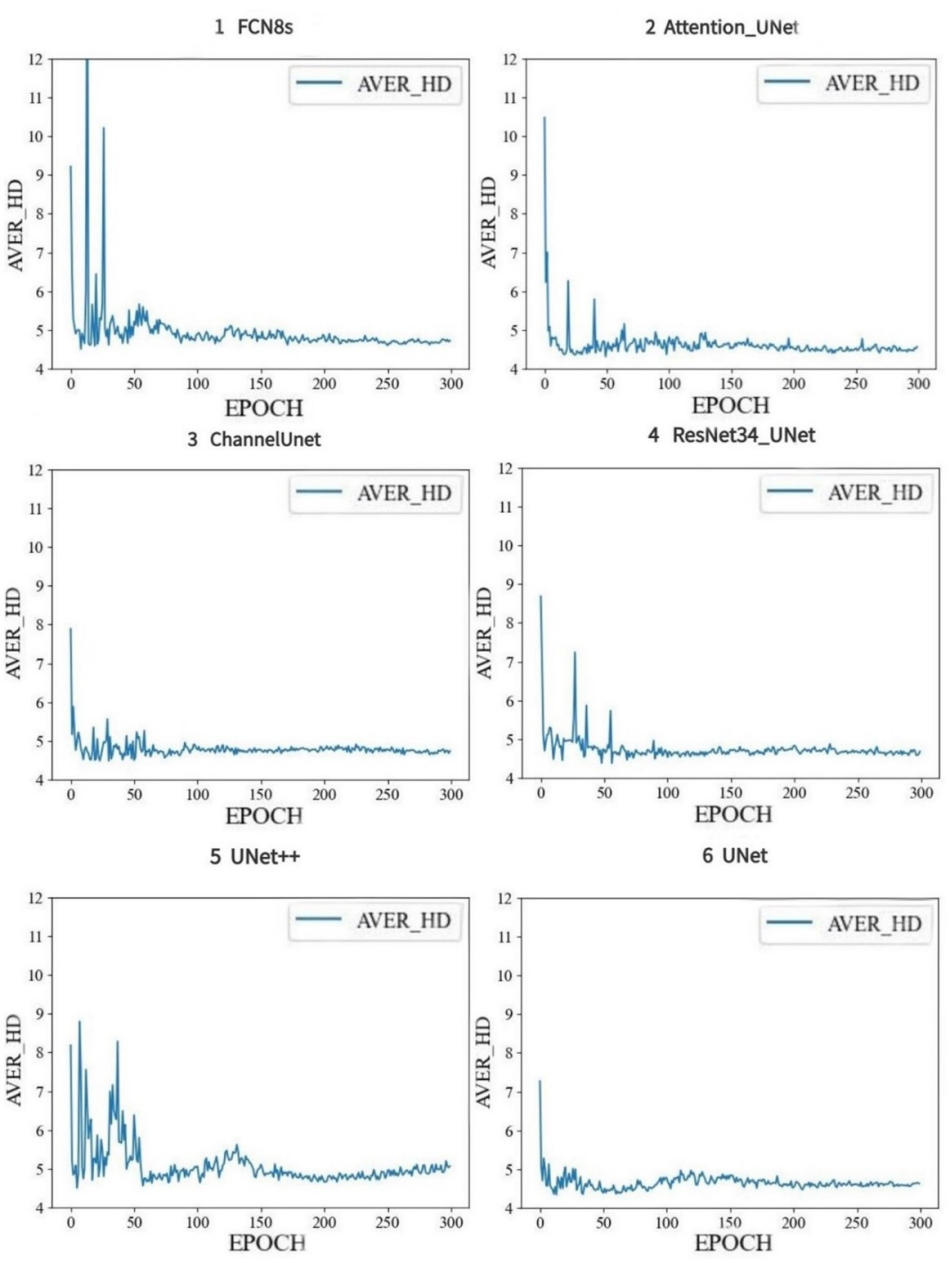
AVER_HD results of six deep learning models.

The AVER_HD index reflects the closeness between the estimated value and the real value, the curves of AVER_HD value from the six models during training were shown (Fig 9). All AVER_HD values generated by the six models approached to stable values, the minimum value of AVER_HD was generated by Attention-UNet is approaching to 4.6, which is also the best value among the six results, while the maximum value was generated by FCN8s is approaching to 4.9, and differences among results of AVER_HD from Channel-Unet, ResNet34-Unet, UNet++ and UNet were very slight.

**Fig 10:**
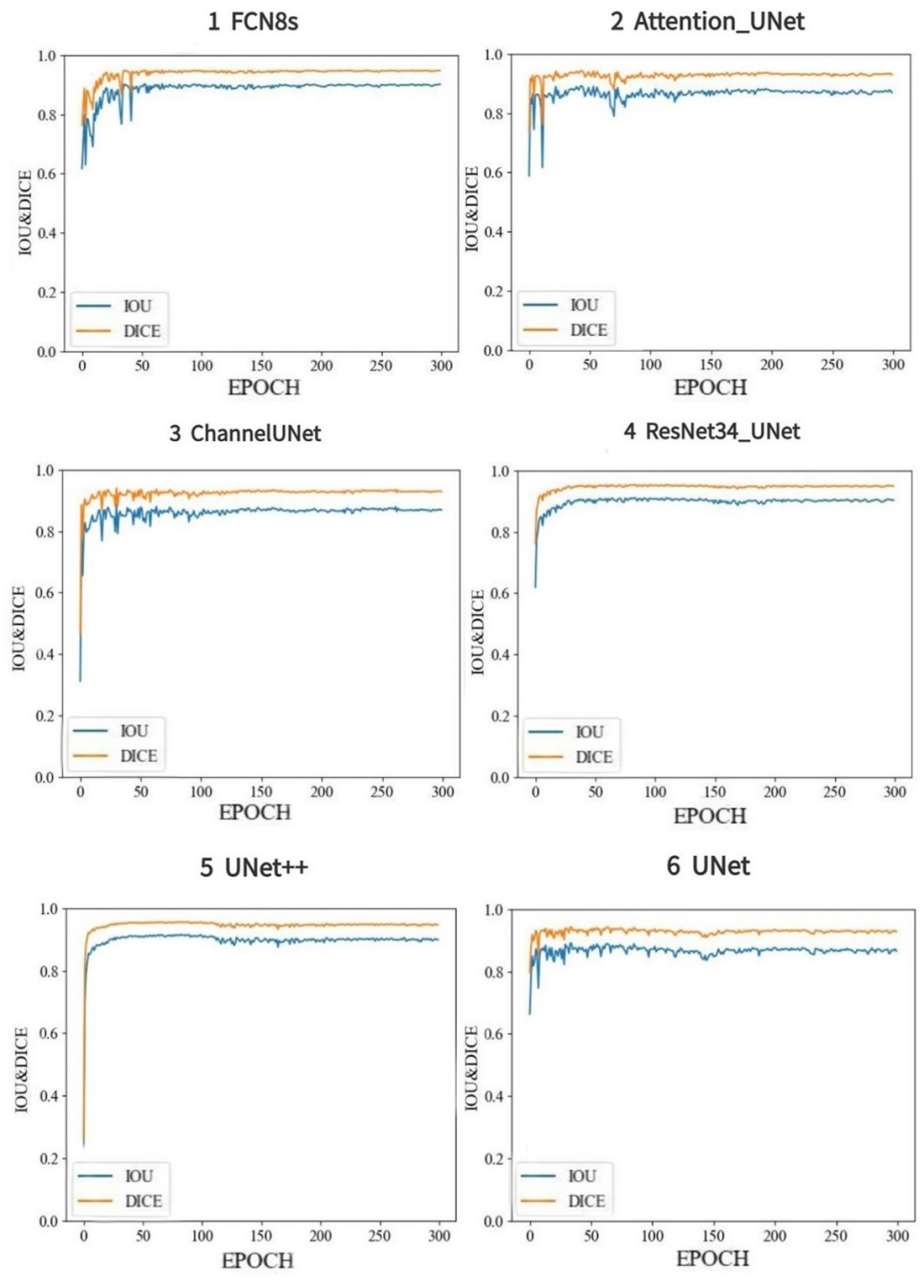
MIOU and DICE of six deep learning models.

MIOU indicates the average predictive segmentation accuracy and DICE represents the similarity between the predictive value and the real value. Results of MIOU and DICE were generated by the 6 models during training process were shown (Fig 10). From the figures, we can find that the MIOU and DICE values from the 6 models quickly reached equilibrium state in training phase, and all of the MIOU values were much larger than 0.87 and all DICE values were more than 0.93, which indicated that the predictive accuracy and the similarity between the predictive value and the real value in image segmentation were high, and deep learning image segmentation were valid. For each model, Attention-UNet produced the highest values of MIOU (0.900±0.012) and DICE (0.947±0.007), which means that Attention-UNet outperformed other methods.

## Discussion

Current challenges in the application of CT imaging technology in the livestock science are: (1) The significant differences in body size between animals pose difficulties in conducting whole-body CT scans on large animals like cattle and horses due to limitations of CT equipment. (2) Manual annotation of data sets by experts will bring uncertainty in results because these results are influenced by expertise, proficiency and skills of the expert. (3) There are notable variations in the boundary limits of organs and tissues in CT images across species, and no segmentation model exists that can consistently achieve optimal results for CT images of different species.

The application of CT image recognition technology to measure animal phenotypic traits is emerging as a new approach to data acquisition [30, 31]. Compared to the application to rabbit and tilapia trait data collection, using CT image technology to measure sheep’s phenotypic traits has a broader application prospect and greater economic benefits. Image segmentation is an important part in CT image 3D imaging, and the segmentation affects the imaging results, which ultimately influences the accuracy of the measurement. Deep learning image segmentation technology is an emerging technology in recent years. Compared with traditional segmentation technologies such as threshold and clustering, deep learning has shown higher performance in intelligence and segmentation accuracy [32].

FCN is a typical deep learning model and its basic structure is a stack of convolutional and pooling layers, following by a series of fully connected layers and a classification layer. However, the disadvantage of FCN is that it requires large data set for the training process and reduces the image size in the convolution and pooling operations. In order to overcome the defects, UNet neural network introduces the adaptive and expansion semantic segmentation to FCN, and uses the encoder-decoder network model to couple FCN to a layer called deconvolution network to improve predictive accuracy [20]. Among the results of evaluation functions on sheep CT image segmentation from different models in this study, the results of FCN are generally worse than those of other models, which are determined by the structure of FCN model.

Although the UNet deep learning model has better performance than the FCN model, the complexity of the network increases during to the layered stack of the encoder or decoder in UNet, and the difference of the input image also increases the complexity of the neural network, all of which have directly impacts on the running time. Longer running times in Table 2 proved it. For each model, the computing resources required were different in the training stage, the increase of network complexity requires more computing resources, which resulting in different running time.

To have comprehensive evaluations on the effects of the six deep learning models, we applied AVER_HD, MIOU, DICE, LOSS to assess deep learning models in experiments. When parameters were set to same values, Attention-UNet, which combined attention mechanism to UNet model generated the best AVER_HD among the six network models (as shown in Table 2) because attention mechanism can improve the efficiency of feature learning and make the fusion strategy better. For MIOU and DICE, Attention-UNet outperformed other methods with the results of 0.900±0.012 and 0.947±0.007, respectively, which indicates that the attention-gate mechanism plays an important role in focusing on important features and inhibiting unnecessary features. In particular, the switch to soft-attention in the skip join and up-sampling module increased the ability of image feature extraction and fusion [33].

Channel-UNet can effectively reduce the LOSS of neural network training and prediction. Owing to the addition of spatial channel convolution operation in the sampling module, Channel-UNet can effectively reflect the pixel information in the high-level feature map, which can achieve tight fusion of multi-scale features and reduce the training LOSS at the same time.

For running time, ResNet34-UNet used the shortest running time to complete the deep learning implementation, it took 22.078±0.368h to complete 300 iterations training. In ResNet34, the feature extraction layer in UNet was replaced with lightweight network to ensure the maximum integration of deep semantic features and combined a large number of skip connections to improve the convergence of image segmentation, these strategies can effectively reduce the problem of gradient disappearance in UNet, and shorten its running time.

For the aforementioned CT image segmentation methods applied to the Loin CT image of sheep, the algorithms can segment the Loin of sheep efficiently, especially when the attention mechanism or ResNet [34] were combined to the network [23]. When VGG16 [35] and other popular lightweight feature extraction networks are used to replace the sub-sampling stage of UNet for feature extraction, higher accuracies can also be achieved.

To improve the performance of deep learning image segmentation, high-quality mask files based on input information to improve the training the training process and enhancing the model’s were proposed [36], the experimental results demonstrate its remarkable self-learning capability and excellent image segmentation performance, the model exhibited significant potential in CT image segmentation, these merits are very helpful to our future research. Despite certain limitations apparent in the application of deep learning CT image segmentation in animal science, these challenges can be gradually overcome with model improvement, data accumulation and upgradation of CT scanning equipment. Deep learning-based image segmentation methods hold substantial promise in CT image applications, which will promote the development of diagnosis of animal diseases and non-destructive measurement of farm animal.

## Conclusion

Six kinds of deep learning model were used to conduct Loin CT image segmentation of meat sheep, which was a key process required to estimate the volume of loin in living sheep and help making selection in breeding program. Four kinds of evaluation index were used to assess results of the image segmentation. Experimental results indicated that Attention-UNet outperformed others methods on Pixel Accuracy, AVER_HD, MIOU and DICE, which were 0.999±0.009, 4.591±0.338, 0.90±0.012 and 0.95±0.007, respectively. These findings demonstrated the high performance of Attention-UNet in segmenting Loin area in CT images.
